# Fibrinolysis-resistant carbonylated fibrin detected in thrombi attached to the vascular wall of abdominal aortic aneurysms

**DOI:** 10.1038/s41598-020-77582-1

**Published:** 2020-11-26

**Authors:** Yuko Suzuki, Hiroki Tanaka, Takahiro Horinouchi, Hideto Sano, Naoki Honkura, Naoki Unno, Soichi Miwa, Tetsumei Urano

**Affiliations:** 1 Department of Medical Physiology, Hamamatsu University School of Medicine, Hamamatsu, Japan; 2 Department of Cellular Pharmacology, Graduate School of Medicine, Hokkaido University, Sapporo, Japan; 3 The Second Department of Surgery, Hamamatsu University School of Medicine, Hamamatsu, Japan; 4 Toyooka Hospital, Toyooka Public Hospitals’ Association, Toyooka, Japan

**Keywords:** Cardiovascular biology, Cardiovascular biology, Circulation

## Abstract

In this study, we investigated how carbonylation of fibrinogen by acrolein modified its indispensable function to enhance fibrinolysis after being converted to fibrin and contributed to generating a fibrinolysis-resistant fibrin clot. Acrolein-treated fibrinogen was subjected to tissue plasminogen activator-induced fibrinolysis assay and the effect of lysine residue carbonylation in fibrinogen on fibrinolysis was analyzed. The acrolein-treated fibrinogen-derived fibrin clot appeared more resistant to fibrinolysis and the N-acetyl 3-formyl-3,4-dehydropiperidino (FDP)-Lysine levels in the lysed solution were positively correlated with the duration of clot lysis. The lysine analog 6-amino hexanoic acid (6AHA), which mimics the C-terminal lysine of fibrin, was carbonylated and its enhancing effect on Glu_1_-plasminogen activation was evaluated. After incubation with acrolein, 6AHA was converted to N-acetyl FDP-6AHA, losing its ability to enhance Glu_1_-plasminogen activation. These results suggest that fibrinogen carbonylation by acrolein to generate N-acetyl FDP-Lysine resulted in the generation of fibrinolysis-resistant fibrin by attenuating the C-terminal lysine-dependent activation of the Glu_1_-plasminogen. In abdominal aortic aneurysms, fibrin(ogen) containing the acrolein adduct N-acetyl FDP-Lysine was detected in the vascular wall-attached thrombi. These results suggest that this mechanism is likely involved in the modification of fibrinolysis-resistant thrombi and to their persistence for a long period.

## Introduction

Acrolein, a highly reactive unsaturated aldehyde, is a well-known pollutant present in air and cigarette smoke^1^, which is also produced in the body as a lipid peroxidation product^2^. Acrolein causes a variety of adverse effects on normal physiological functions, including those of the cardiovascular system^3^, through diverse mechanisms such as the induction of oxidative, mitochondrial-, and ER- stresses, and its adduction to protein and DNA^3^. Altogether, these mechanisms of acrolein either indirectly or directly damage cellular functions, modify protein/DNA functions, and elicit various pathological outcomes. For the formation of acrolein adducts with proteins, the sulfhydryl group of cysteine, the imidazole group of histidine, and the amino group of lysine are targeted.

Under oxidative stress, carbonylation by acrolein involves conjugation of acrolein to the amino acid group at the side chain of lysine residues in target proteins^4^ and the pathological indications of this process have been analyzed^2,5^. Among plasma proteins, the conjugation of acrolein was found to be most abundant in albumin^4^ and the levels of protein-conjugated acrolein were associated with the incidence of ischemic stroke in a mouse model^6^ and with silent brain infarction and carotid atherosclerosis in human^4^.

Fibrinogen, a soluble plasma protein and the source of thrombi, is also known to be carbonylated in post-myocardial infarction patients^7^. As a result, carbonylated fibrinogen in the patients’ plasma increased almost 3.5 fold in average, although the magnitude of the increase was depending on the severity of the oxidative stress^7^. As in case of other proteins, carbonylation modified the functions of fibrinogen and its thrombin-catalyzed product, fibrin. Carbonylated fibrinogen purified from patients plasma showed reduced clotting ability and decreased susceptibility to plasmin-dependent degradation, though the precise underlying mechanism has not yet been clarified^7^. For effective fibrinolysis, the formation of a trimolecular complex consisting of the plasminogen and the tissue plasminogen activator (tPA) on the fibrin surface is essential. The resulting conformational change of Glu_1_-plasminogen after binding to the C-terminal lysine of fibrin through its lysine binding sites (LBSs), as well as the template mechanism, is responsible for effective tPA-mediated activation of the Glu_1_-plasminogen^8^. Thus, the modification of lysine residues in fibrin(ogen) may impair the effective activation of Glu_1_-plasminogen on the fibrin surface and the subsequent fibrinolysis.

Fibrinolysis-resistance makes thrombi retained longer time, which is causative of many ischemic disorders. Prolonged retention of thrombi in the vascular wall is frequently observed (70–80%) in abdominal aortic aneurysm (AAA)^9^, and triggers many pathological events which lead to derangements of vascular function and configuration. Though oxidative stress and/or smoking are believed causative to develop AAA and to the associated thrombi in the vascular wall, the details of the mechanism of the prolonged retention of thrombi are not clarified yet.

In the present study, we analyzed how fibrinogen carbonylation modifies fibrin-dependent enhancement of plasminogen activation and fibrinolysis. We found that the clot generated by acrolein-treated fibrinogen was more resistant to fibrinolysis compared to that generated by non-treated fibrinogen. Moreover, the lysine analog 6-amino hexanoic acid (6AHA), which mimics the role of the C-terminal lysine of fibrin, lost its ability to enhance Glu_1_-plasminogen activation after acrolein treatment. Further, we successfully demonstrated the existence of acrolein-conjugated fibrin in the thrombi attached to the vascular wall of AAA in surgically resected specimens.

## Results

### Effect of cigarette smoke extract (CSE) treatment of fibrinogen on fibrin clot lysis time and N-acetyl FDP-lysine generation

**Figure 1:**
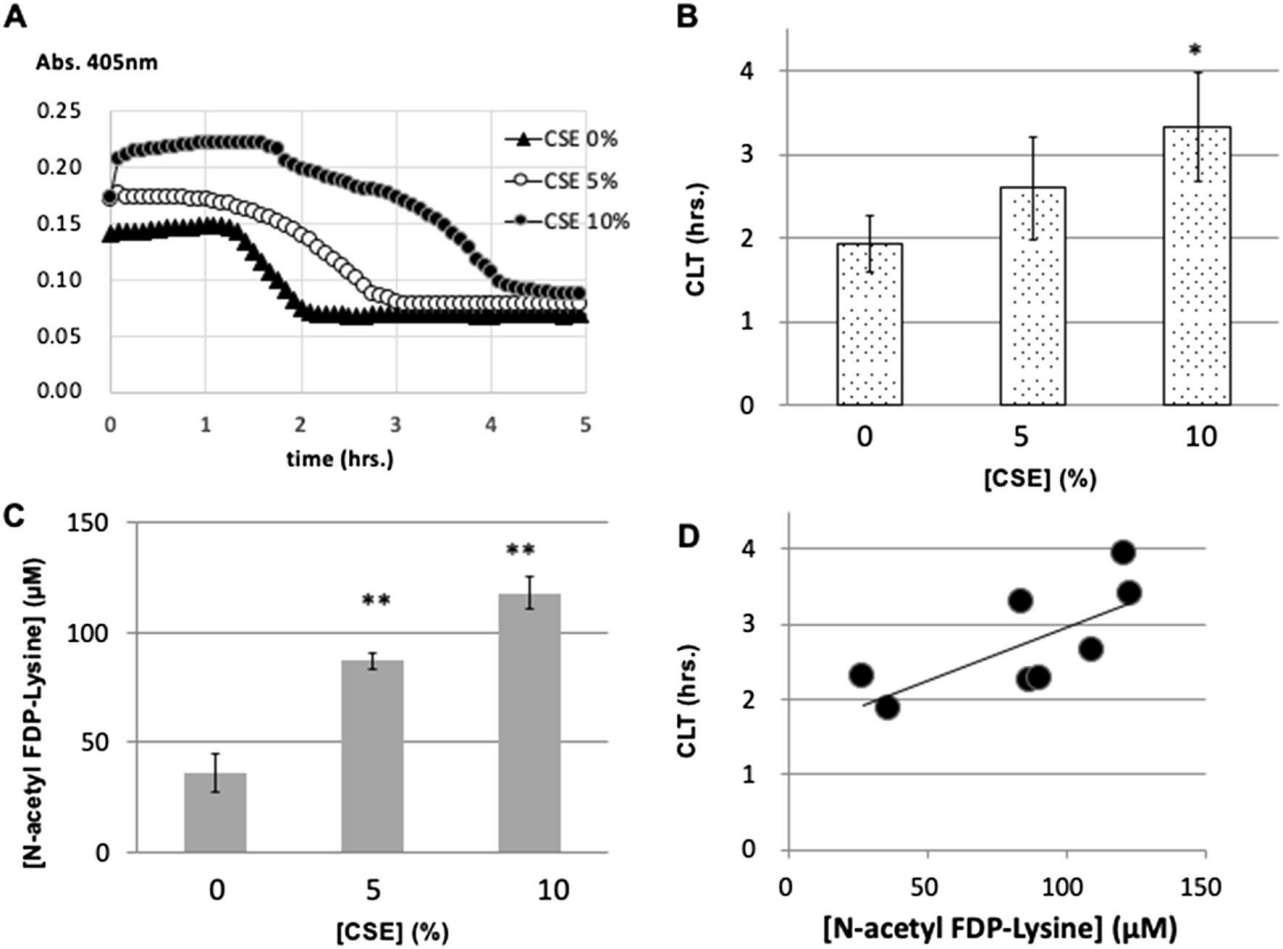
Effect of CSE treatment of fibrinogen on fibrin clot lysis time and N-acetyl FDP-Lysine generation. (**A**) Original trace of clot lysis time of fibrin clot formed by CSE-treated fibrinogen at different concentrations. Y axis shows absorbances at 405 nm wave length and X axis shows time (hours). (**B**) Clot lysis time of fibrin clot formed by CSE-treated fibrinogen at different concentrations. N = 3. (**C**) Concentration of N-acetyl FDP-Lysine in the lysed solution of fibrin clot formed by CSE-treated fibrinogen at different concentrations. N = 3. (**D**) Correlation between clot lysis time and the concentration of N-acetyl FDP-Lysine in the lysed solution was analyzed by Pearson’s correlation coefficient (r = 0.75, *p* = 0.018). Dose-dependencies were evaluated by the Williams multiple comparison test (*p < 0.05, **p < 0.01).

Human fibrinogen was treated with CSE at different concentrations and was used for tPA-induced clot lysis assay. The time taken for clot lysis was longer when CSE-treated fibrinogen was employed (Fig. 1A,B). After achieving total clot lysis, we also measured the amount of N-acetyl FDP-Lysine in the lysed solution. The concentration of N-acetyl FDP-Lysine was higher in the CSE-treated fibrinogen according to the concentration of CSE (Fig. 1C) and showed a positive correlation with the clot lysis time (Fig. 1D).

### Effects of carbonylation of fibrinogen by acrolein on clot lysis time

**Figure 2:**
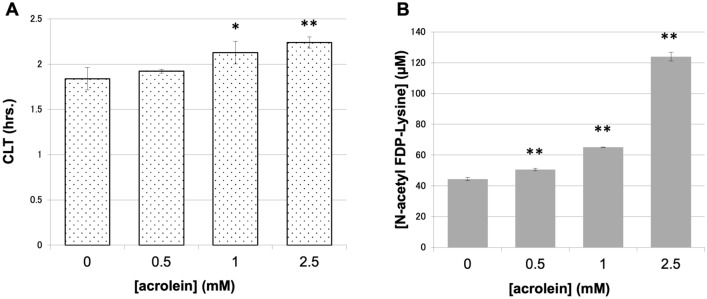
Influence of carbonylation of fibrinogen by acrolein on clot lysis time. (**A**) Clot lysis time of fibrin clot formed by acrolein-treated fibrinogen at different concentrations. N = 3. (**B**) Concentration of N-acetyl FDP-Lysine in the lysed solution of fibrin clot formed by acrolein-treated fibrinogen at different concentrations. N = 3. Dose-dependencies were evaluated by the Williams multiple comparison test (*p < 0.05, **p < 0.01).

Human fibrinogen was treated with acrolein at different concentrations and was used for tPA-induced clot lysis assay. The time taken for clot lysis was longer when acrolein-treated fibrinogen was used in an acrolein-concentration dependent manner (Fig. 2A). The concentration of N-acetyl FDP-Lysine in the lysed solution of acrolein-treated fibrinogen was higher than that in non-treated fibrinogen (Fig. 2B). Although we tried to obtain higher amounts of carbonylated fibrinogen, fibrinogen aggregated after incubation with concentrations of acrolein higher than 2.5 mM, as was observed for other proteins^10^, and could not be used for clot lysis assay.

These results suggested that the fibrin clot formed by acrolein-treated fibrinogen was more resistant to tPA-induced clot lysis, most likely due to a modification of lysine residues in the fibrinogen molecule and the associated reduction of plasminogen binding to fibrin.

### Effects of carbonylation of 6AHA by acrolein on its enhancement of Glu1-plasminogen activation

**Figure 3:**
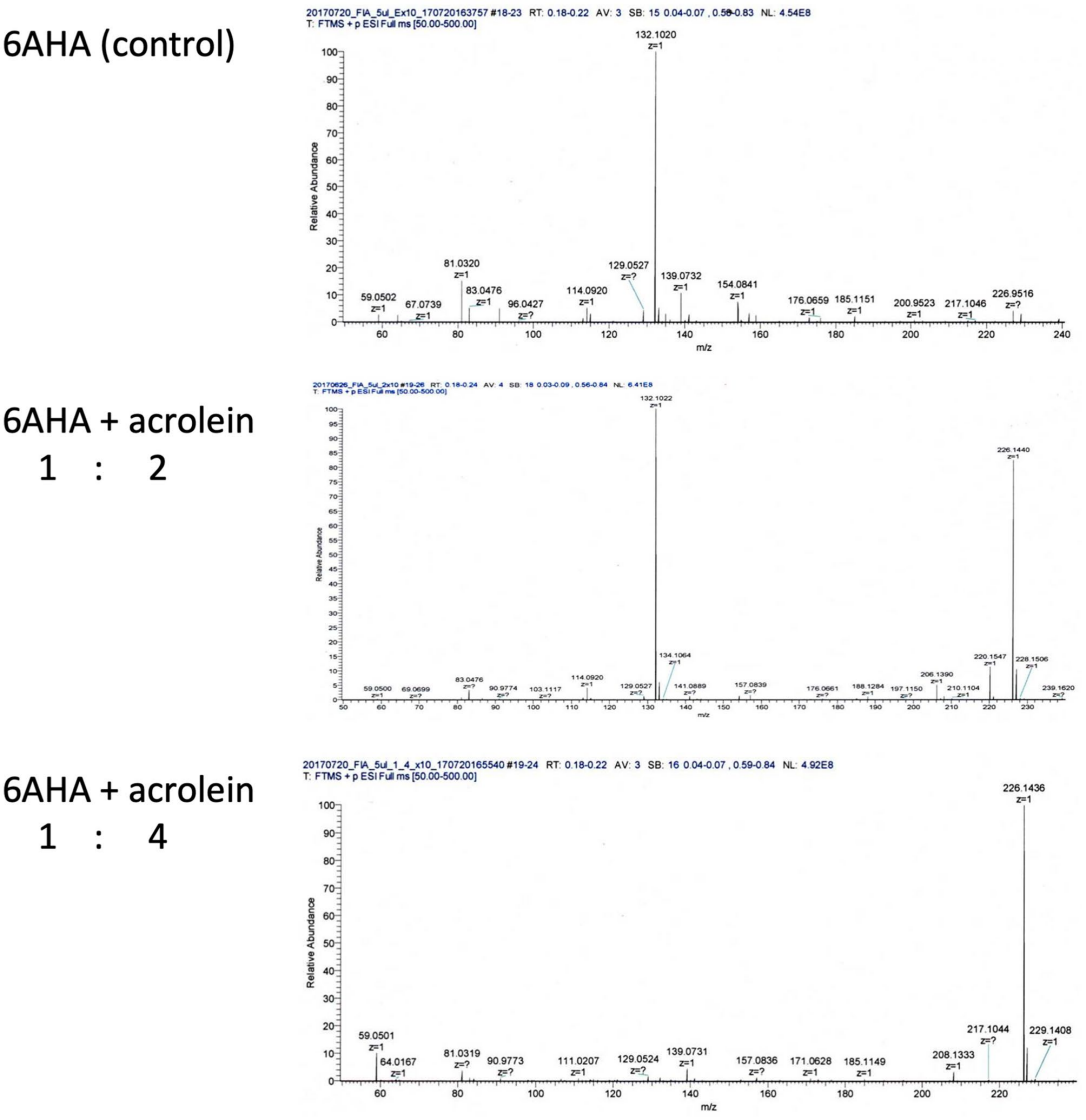
Carbonylation of 6AHA by acrolein. After the treatment of 6AHA at the molar ratios of 1:2 and 1:4, the generation of N-acetyl FDP-6AHA was analyzed via Orbitrap LC–MS. An additional peak in the 6AHA band (132.1020 m/z), corresponding to N-acetyl FDP-6AHA (226.1440 m/z), was observed after the treatment at a molar ratio of 1:2. Only an N-acetyl FDP-6AHA (226.1440 m/z) peak was observed after the treatment at a molar ratio of 1:4.

**Figure 4:**
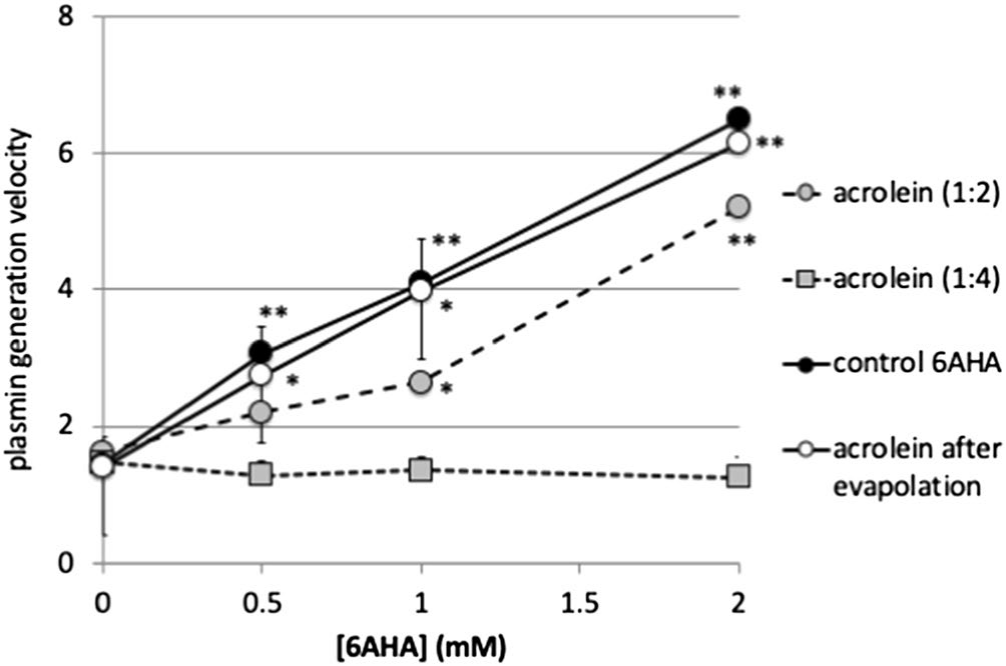
Loss of enhancing effect of 6AHA on uPA-catalyzed Glu_1_-plasminogen activation after conversion to N-acetyl FDP-6AHA. 6AHA enhanced uPA-catalyzed Glu_1_-plasminogen activation in a dose-dependent manner (control 6AHA). After incubation with acrolein at different molar ratios (1:2 and 1:4), 6AHA lost its enhancing effect on Glu_1_-plasminogen activation in an acrolein concentration-dependent manner. When 6AHA was incubated with acrolein solution after evaporation, however, Glu_1_-plasminogen activation was enhanced in a dose-dependent manner as was control 6AHA.

Treatment of 6AHA by excess amounts of acrolein successfully led to the carbonylation of 6AHA medially or almost fully after incubation at the molar ratios of 1:2 and 1:4, respectively (Fig. 3). 6AHA enhanced urokinase-type plasminogen activator (uPA)-catalyzed Glu_1_-plasminogen activation in a dose-dependent manner (Fig. 4), as has been reported previously^11^. However, 6AHA lost its enhancing effect on Glu_1_-plasminogen activation according to the extent of carbonylation, although 6AHA mixed with acrolein solution after evaporation exhibited a similar enhancement of Glu_1_-plasminogen activation to that by control 6AHA, which was incubated with the solvent alone. This enhancement is explained by a conformational change in Glu_1_-plasminogen from a closed form to a loose form^11^, as a result of the dissociation of intra-molecular binding between Lys_50_ in its pan-apple domain and a LBS in kringle 5^8,12,13^. Carbonylation clearly modified the binding capacity of 6AHA to the LBS in kringle 5.

### Identification of carbonylated lysine in the thrombi attached to the vascular wall of AAA

**Figure 5:**
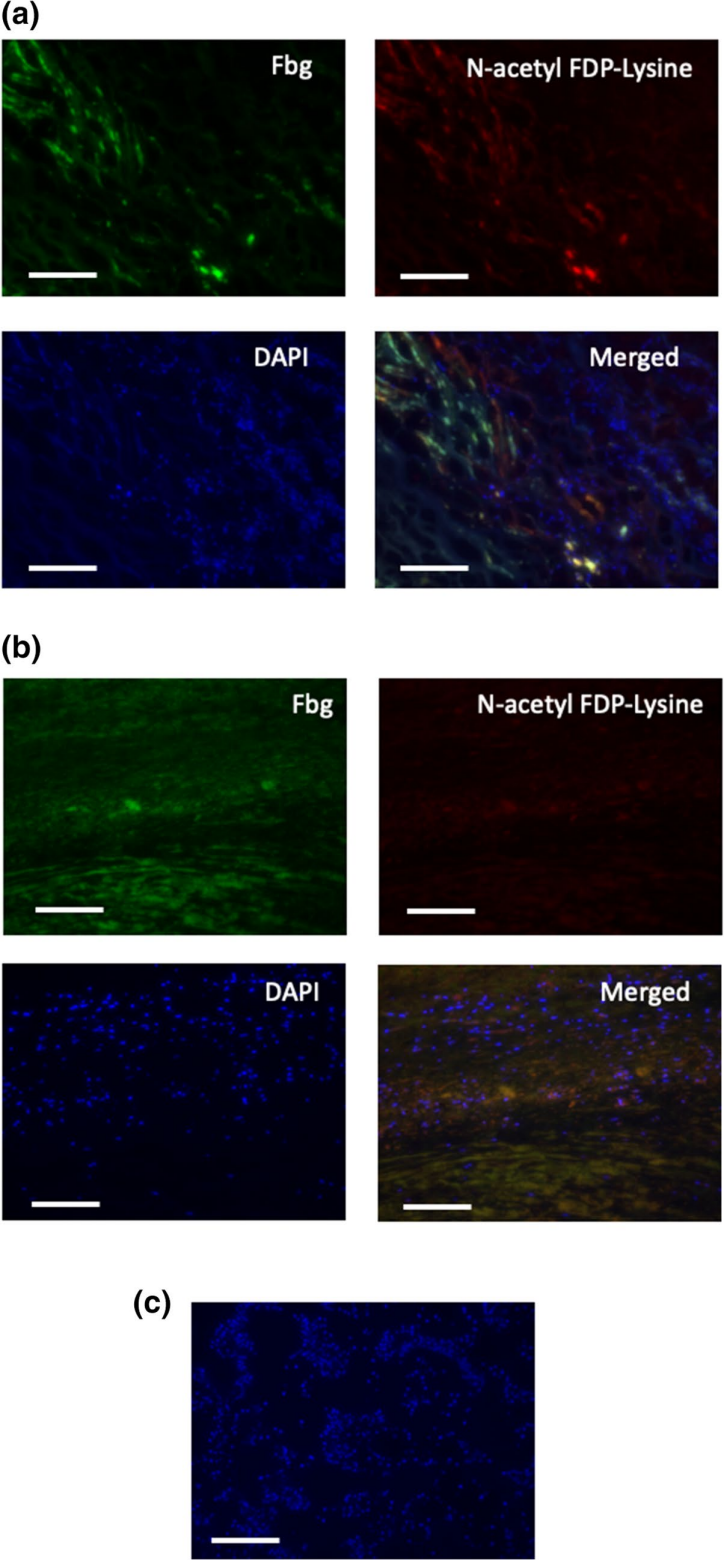
Identification of carbonylated lysine in the thrombi attached to the vascular wall of AAA and control. Surgically resected specimen of AAA vascular wall (**a**) and control vascular wall (**b**) were subjected to immunostaining by anti-human fibrinogen (green) and anti-N-acetyl FDP-Lysine (red), respectively. Similar positive staining patterns were observed (merged), though the staining by anti-N-acetyl FDP-Lysine was stronger in AAA vascular wall. Nuclei are counterstained with DAPI. In (**c**), image was obtained with direct application of the secondary antibodies and nuclei are counterstained with DAPI. Scale bar shows 200 µm.

**Figure 6:**
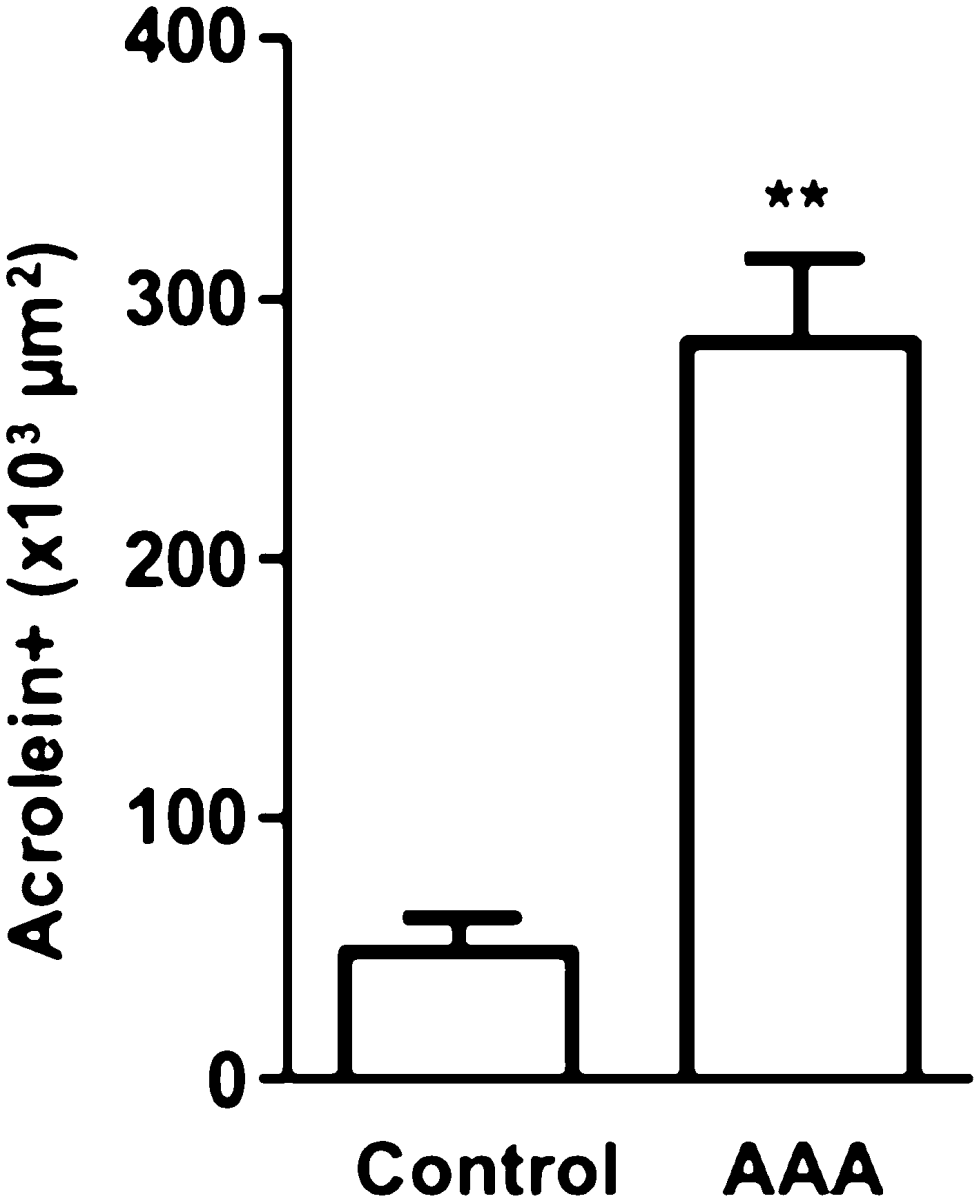
Area of carbonylated lysine positive staining in the vascular wall of normal and AAA aorta. Surgically resected specimen of the normal aorta (N = 5, M/F = 5/0) and AAA vascular wall (N = 9, M/F = 9/0) were subjected to immunostaining by anti-N-acetyl FDP-Lysine. The area of positive staining in AAA was significantly larger than that in the normal aorta. **Unpaired t-test, *p* < 0.001.

Using surgically resected specimens, we examined the existence of fibrin containing carbonylated lysine in vascular wall-attached thrombi. Both patchy and fibrous staining by anti-N-acetyl FDP-Lysine antibody were observed in the thrombi, most of which were also detected using the anti-human fibrinogen antibody (Fig. 5a). The colocalization of positive staining by these two antibodies suggests that N-acetyl FDP-Lysine residues detected in the thrombi were of fibrin origin. When similar vascular wall specimens obtained from control patients were stained (Fig. 5b), the positive staining signal was significantly weaker than that observed for the AAA vascular wall (Fig. 6). Image obtained with direct application of the secondary antibodies was used as control in which nuclei were counterstained with DAPI (Fig. 5c).

## Discussion

In this study, we proved that acrolein contained in the CSE modified fibrinogen more resistant to fibrinolysis after being converted to fibrin by the generation of N-acetyl FDP-Lysine. We also found that fibrin containing N-acetyl FDP-Lysine existed in the thrombi attached to the vascular wall of AAA patients. These results suggest that carbonylation of fibrin(ogen) may contribute to the prolonged retention of thrombi at the vascular wall of AAA by making fibrin more resistant to fibrinolysis. This is the first report to demonstrate a modification of the well-known function of a protein due to the acrolein-dependent modification of the lysine residues in the protein.

Cigarette smoking is strongly related to the pathogenesis of cardiovascular diseases^14^. Endothelial cell dysfunction and atherosclerotic plaque formation, fragile plaque formation, and thrombus formation are all associated with smoking. For thrombus formation, besides thrombogenicity due to a hypercoagulable state and enhanced platelet activation, less effective fibrinolysis also plays a role^14^. Impaired tPA secretion from vascular endothelial cells^15^ and increased plasminogen activator inhibitor type 1 (PAI-1) levels^16^ are well-known factors that lead to suppressed fibrinolysis in smokers^14^. In the present study, we demonstrated another possible mechanism for smoking to cause the impairment of fibrinolysis, wherein the acrolein adduction to lysine residues increases the resistance of fibrinogen to fibrinolysis after its conversion to fibrin.

The fibrinolytic system is spatiotemporally finely regulated via sophisticated mechanisms. However, the basic mechanism is rather simple. The generated fibrin is quickly dissolved through trimolecular complex formation by the gathering of both tPA and plasminogen on the fibrin surface^17^. The binding of plasminogen to fibrin is mediated by the interaction between the C-terminal lysine of partially digested fibrin and LBSs in kringle domains of Glu_1_-plasminogen, which facilitate tPA-catalyzed Glu_1_-plasminogen activation through both a template mechanism and conformational change of Glu_1_-plasminogen^8,17^. The physiological relevance of this mechanism is proved by the strong inhibitory effect of lysine analogs, including 6AHA and tranexamic acid, on fibrinolysis^18^ and the effective suppression of bleeding after trauma caused by hyperfibrinolysis^19^.

The conformational change in Glu_1_-plasminogen induced by C-terminal lysine of fibrin is easily mimicked by lysine analogs. 6AHA enhances PA-catalyzed Glu_1_-plasminogen activation by converting its tight conformation to a looser form by dissociating intra-molecular binding between Lys_5o_ in the Pan-Apple domain and LBS in the kringle 5 domain^12,13,18,20^, which was also shown in the present study. N-acetyl FDP-6AHA, however, lost such activity most likely caused by a loss of positive charge of the amino residue, which is essential for binding to the LBS binding pocket in kringle 5^8,13^. Fibrin clot formed by CSE-treated fibrinogen was more resistant to tPA-catalyzed fibrinolysis. The clot lysis time showed a positive correlation with the amounts of N-acetyl FDP-Lysine in the lysed solution. Similar results were obtained by acrolein-treated fibrinogen. The clot formed from acrolein-treated fibrinogen was more resistant to fibrinolysis, and the amount of N-acetyl FDP-Lysine in the lysate was higher compared to control fibrinogen. These results suggest that acrolein treatment increased the contents of N-acetyl FDP-Lysine in the fibrinogen molecule, which made the generated fibrin more resistant to fibrinolysis by attenuating plasminogen-binding to its surface. Exposure to CSE appeared enough for fibrinogen to get carbonylated by acrolein and become more resistant to fibrinolysis after being converted to fibrin. These may explain the involved mechanisms elucidated in previous findings in the thrombotic patients. One of these mechanisms is the impaired plasminogen binding to venous thrombus, found in patients with venous thrombosis who had higher total carbonylated protein in their plasma^21^. The other is the decreased susceptibility to plasmin-dependent fibrinolysis of carbonylated fibrinogen obtained from patients with myocardial infarction^7^.

Inadequate thrombus formation causes both arterial and venous thrombosis. Intraluminal thrombi attached to the vascular wall are frequently observed (70–80%) in AAA^9^. Dysfunction of normal vascular endothelial cells due to atherosclerosis, increase in thrombogenic factors, including fibrinogen and PAI-1 due to hyperlipidemia, and disruption of laminar flow and creating turbulent flow at the widened vascular bed, frequently seen in AAA, are all risk factors for thrombus formation. Resistance to thrombolysis is another factor for thrombi remaining in the vasculature, as continuous fibrinolysis seems to occur in the wall-attached thrombi, especially at the abluminal side^22^. A dysfunction of normal vascular endothelial cells to keep high fibrinolytic activity by secreting and retaining tPA on their surface and a suppression of fibrinolytic potential due to the increase in plasma PAI-1 level under many pathological conditions including inflammation, obesity, hyperlipidemia, and aging are considered to impair fibrinolysis and cause thrombogenicity^17^. The impairment of fibrinolysis due to acrolein adduction to lysine residues of fibrin appeared to be another mechanism involved in thrombi remaining on the vascular wall of AAA. Retained intraluminal thrombi are known to initiate local hypoxia and the associated inflammation, which results in the enlargement of AAA^9^. Thus in addition to triggering the development of AAA through the obstruction of vasa vasorum as our group suggested^23–25^, hypoxia induced by either atherosclerosis or thrombus formation appeared deteriorate AAA pathology.

Smoking is an important risk factor for AAA^9^, and  the water-soluble fraction of cigarette smoke contains acrolein^1^. In smokers, significantly increased levels of oxidized plasma proteins including carbonyl-containing fibrinogen compared to non-smokers are reported^26^. Acrolein is also produced in our body as a lipid peroxidation product, and its conjugated proteins are used as markers of oxidative stress^2^. In the AAA vascular wall as well as its associated thrombi, many pathological events occur due to tissue hypoxia and/or regional inflammation, and oxidative stress is generated by infiltrated polymorphonuclear leukocytes^27^. In addition to acrolein liberated from cigarette smoke, those generated at the inflammatory site of AAA seem to contribute in generating carbonylated fibrin. As intraluminal thrombus is known to accelerate inflammation and AAA enlargement, strategies to render the thrombi more susceptible to fibrinolysis and to facilitate its removal should be considered to improve pharmacological treatments provided to patients. Antioxidants have been speculated to be such candidates for a long time^28^, and were recently highlighted in our animal model^23,29^.

In conclusion, the present study demonstrated that acrolein in CSE carbonylated fibrinogen such that fibrinogen was not quickly dissolved after conversion to fibrin. The extent of the carbonylation of lysine residues in the fibrinogen molecule was well-correlated with fibrinolysis impairment. These results explain the merit of measuring carbonylated protein levels in plasma to evaluate the risk of thromboembolic diseases^6,7,30^.

## Materials and methods

### Chemicals

The following materials were purchased from the indicated sources: Acrolein monomer (Tokyo Chemical Industry, Co. Ltd., Japan), 6AHA (Sigma-Aldrich Japan Co. LLC), S-2251 (Chromogenix, Mölndal, Sweden), Acrolein-Lysine Adduct Competitive EIA Kit (Takara Bio-Com, Japan), human fibrinogen (Enzyme Research Laboratory, South Bend, IN, USA), human thrombin (Benesis, Osaka, Japan), urokinase-type plasminogen activator (uPA) (Mitsubishi Pharma Corporation, Osaka, Japan), anti- FDP-Lysine antibody (NIKKEN SEIL Co, Shizuoka, Japan) and goat anti-human fibrin(ogen) antibody (GeneTex, Inc., Irvine, CA, USA). Human recombinant tPA (TD2061) was kindly provided by TOYOBO CO., LTD., Osaka, Japan. Human Glu_1_-plasminogen was purified from fresh-frozen human plasma (Japanese Red Cross Society) using lysine sepharose. CSE was prepared using a previously described continuous smoking protocol^31^, which contains acrolein at a concentration of approximately 3.4 mM^32^.

### Carbonylation of 6AHA and fibrinogen

In tightly sealed tubes, 6AHA (50 mM) was incubated with different concentrations of acrolein monomer (0, 100, and 200 mM) at 60 °C overnight and the mixture was left in a draft chamber for 30 min after removing the seal to evaporate the remaining acrolein^10^. The mixture was kept at − 70 °C for use in plasminogen activation assay and mass spectroscopy (MS) analysis.

Fibrinogen solutions (22 µM) were incubated with different concentrations of acrolein monomer (0, 0.5, 1.0, 2.5 mM) at 37 °C overnight and subjected to clot lysis assay. A fraction of the lytic solution was subjected to N-acetyl FDP-Lys ELISA assay.

### Mass spectroscopy

The formation of N-acetyl FDP-6AHA by the addition of acrolein to 6AHA was analyzed using Orbitrap LC–MS (Qexactive: Thermo Scientific) with flow injection analysis.

### Plasminogen activation assay

The mixture of Glu_1_-plasminogen (0.5 µM), uPA (1.0 nM), and S2251 (0.4 mM) was incubated with different concentrations of either acrolein treated- or non-treated-6AHA in 96-well microtiter plates at 37 °C and the absorbance in each well at 405 nm wavelength was measured at intervals of one minute. Initial plasmin generation velocity was calculated from the slope of the absorbance vs. time^2^ plot^20,33^.

### Clot lysis assay

Glu_1_-plasminogen (0.5 µM), tPA (0.02 nM), and human thrombin (1 U/ml) were mixed with either acrolein/CSE treated- or non-treated-human fibrinogen (2.9 µM) in 96-well microtiter plates at 37 °C and the absorbances of the clots formed in the wells were measured at a wavelength of 405 nm every one minute after covering with liquid paraffin. Clot lysis time was defined as the time required for the absorbance to attain the midpoint between the average high values of the fibrin clot and the average low values of the lytic solution^34^.

### Sample collection

We examined the aortic samples from 9 patients who had undergone elective open surgery to repair AAA between April 2014 and March 2015 at the Division of Vascular Surgery, Hamamatsu University School of Medicine, Hamamatsu, Japan. All the patients were male, with a mean age of 78.1 ± 5.3 years, and their aortic tissues and thrombi were intraoperatively removed. Additionally, aorta tissue samples, collected from routine autopsies in the Department of Pathology, Hamamatsu University Hospital, were used as control. All the patients including control patients provided informed consent. We excluded from the study autopsy specimens from patients with collagen disease and/or aortic aneurysm or dissection and conducted our analyses on samples from 5 patients, mean age 73.2 ± 4.1 years old. Tissue samples obtained from autopsies were fixed in formalin. The study was conducted in accordance with the Declaration of Helsinki principles and was approved by the Ethics Committee at Hamamatsu University School of Medicine.

### Immunofluorescence staining

The cellular distribution of N-acetyl FDP-Lysine was analyzed by immunofluorescence staining^24,35^ of the excised tissues using a mouse anti- human N-acetyl FDP-Lysine antibody (1:100) and a goat anti- human fibrin(ogen) antibody (1:100). The slides were then stained with appropriate secondary antibodies and counterstained with 4′,6-diamidino-2-phenylindole (DAPI) to visualize the nuclei. Histological images were analyzed using an inverted microscope (BZX-700, KEYENCE, Osaka, Japan). The intensity of each area was quantified using Scion Image software (Scion, Austin, TX, USA). We set a threshold by which to compute the area positive for N-acetyl FDP-Lysine and then calculated the ratio of the positive area to the total area of each section.　Significant difference in the staining of N-acetyl FDP-Lysine between AAA group and control group was analyzed by Unpaired t-test.

### Statistical analysis

Data were statistically analyzed by Statcel 3, an add-in software for Excel (OMS Publishing Inc., Saitama, Japan). Dose-dependencies were evaluated by the Williams multiple comparison test.
